# Unraveling the role of natriuretic peptide clearance receptor (NPR3) in glomerular diseases

**DOI:** 10.1038/s41598-024-61603-4

**Published:** 2024-05-24

**Authors:** Dina Dabaghie, Emmanuelle Charrin, Pernilla Tonelius, Birgitta Rosengren, Gizem Korkut, Anna B. Granqvist, Mark Lal, Jaakko Patrakka

**Affiliations:** 1 Division of Pathology, Department of Laboratory Medicine, Karolinska Institutet, Huddinge, Sweden; 2 Bioscience Renal, Cardiovascular, Renal and Metabolism (CVRM), R&D Biopharmaceuticals, AstraZeneca, Gothenburg, Sweden; 3 Department of Pathology, Unilabs, Stockholm, Sweden

**Keywords:** Molecular biology, Diseases, Nephrology

## Abstract

Natriuretic peptides (NPs) are cardio-derived hormones that have a crucial role in maintaining cardiovascular homeostasis. Physiological effects of NPs are mediated by binding to natriuretic peptide receptors 1 and 2 (NPR1/2), whereas natriuretic peptide receptor 3 (NPR3) acts as a clearance receptor that removes NPs from the circulation. Mouse studies have shown that local NP-signaling in the kidney glomerulus is important for the maintenance of renal homeostasis. In this study we examined the expression of NPR3 in kidney tissue and explored its involvement in renal physiology and disease by generating podocyte-specific knockout mice (*NPR3*^*podKO*^) as well as by using an NPR3 inhibitor (NPR3i) in rodent models of kidney disease. NPR3 was highly expressed by podocytes. *NPR3*^*podKO*^ animals showed no renal abnormalities under healthy conditions and responded similarly to nephrotoxic serum (NTS) induced glomerular injury. However, NPR3i showed reno-protective effects in the NTS-induced model evidenced by decreased glomerulosclerosis and reduced podocyte loss. In a ZSF1 rat model of diabetic kidney injury, therapy alone with NPR3i did not have beneficial effects on renal function/histology, but when combined with losartan (angiotensin receptor blocker), NPR3i potentiated its ameliorative effects on albuminuria. In conclusion, these results suggest that NPR3 may contribute to kidney disease progression.

## Introduction

Renal glomerular disease processes are the most common cause of chronic kidney disease (CKD) affecting millions of people worldwide^1^. Podocytes, endothelial cells, and mesangial cells are the principal glomerular cell types. They play a unique and specialized role in the maintenance of normal physiology, as well as are the main targets of injury in many forms of CKD^2^. Today, glomerulopathies are treated with drugs that do not target specific glomerular disease mechanisms but are believed to have indirect reno-protective effects (such as via blood pressure).

Natriuretic peptides (NPs) are a family of hormones/paracrine factors that interact with their receptors; a family of peptide binding proteins called natriuretic peptide receptors (NPRs). Most of the physiological effects of NPs have been attributed to NPR1 (NPRA) or NPR2 (NPRB), which, upon NP binding, catalyze the synthesis of intracellular cyclic-GMP (cGMP), a major down-stream effector of NP-signaling pathway. The third member of this receptor family, NPR3 (NPRC), lacks the intracellular signaling domain and acts as a clearance receptor that removes NPs from the circulation and thus suppresses the pathway^3^.

In the glomerulus, NPR1 is highly expressed by podocytes. Inactivation of NPR1 specifically in podocytes does not affect systemic NP levels or result in renal disease. However, under pathological conditions, the absence of NPR1 in podocytes promotes glomerular damage, suggesting that local NP signaling in the glomerulus contributes to renal protection^4^. In addition, a significant downregulation of NPR1 and NPR3 has been observed in the glomerular tissue of diabetic nephropathy (DN) patients^5^. Meanwhile, NPR3 was reported to suppress NPR1-cGMP signaling in heart fibrosis and to enhance the response towards TGF-ß1 signaling^6^. Furthermore, NPR3 locally modulates the availability of NPs at their target organs, as was seen in adipose tissue, suggesting that the activity of NPR3-mediated NP clearance can be tailored to specific physiological needs^7^. High expression of NPR3 is found in the glomerular fraction of the kidney by microarray^5^, suggesting that the clearance receptor may have a role in the maintenance of glomerular homeostasis.

Targeting the NP-system has been a long-term goal in the therapeutic field. Increasing the abundance of NPs either by increasing the circulating NPs via NP administration or through decreased degradation and clearance have been explored. Synthetic infusion of NPs in patients showed beneficial effects, but the short half-life in vivo and the adverse effects limits the use of these agents^3^. The ability to decrease NP degradation via neprilysin inhibitor (NEPi) was only successful in combination therapy with Angiotensin Receptor Blockers (ARBs)^8^. NEPi/ARB treatment was shown to be effective in hypertensive and heart failure patients^8,9^, and this combination has shown reno-protective effects in preclinical models of diabetes and fibrosis^10–12^. Notably however, neprilysin is a pleiotropic enzyme with many substrates beyond NPs^9^, and the individual contribution of changes in the levels of numerous proteins to functional effects is difficult to deconvolute.

Another approach to selectively increase NP levels would be by targeting NPR3 with specific ligands that bind to the receptor and decrease NP clearance. Treatment with NPR3 inhibitor ANP (4–23) in a murine model of focal segmental glomerulosclerosis (FSGS) showed some promising results but faced with the limitation of fast degradation^13^. Other studies have reported selective potent NPR3 inhibitors that showed an increase in cGMP levels in cultured cells and mice^14,15^. Yet, as far as we are aware, no studies have been reported on the effect of these inhibitors in the context of renal protection and CKD development.

We hypothesized that targeting glomerular NPR3 can increase locally NP activity and offer glomerular protection and potentially slow the progression of CKD. In the current study, we test this hypothesis by targeting NPR3 genetically and pharmaceutically in animal models of CKD.

## Material and methods

### Study approval

The Ethical Review Board in Stockholm, Sweden (archive numbers 2010/579-31 and 2016/615-32) has approved the use of human kidney tissue for the study. Informed consent was obtained from all individuals. The design and conduct of our study concerning human samples are compliant with our ethical permits and the Declaration of Helsinki.

Our experimental protocols in mouse were approved by The Ethical Committee for Research Animals, Linköping, Sweden (archive number DNR1336-19, DNR 14071-19). For rat work, our experimental protocols were approved by the Regional Laboratory Animal Ethics Committee of Gothenburg, Sweden (DNR 2327). All methods were performed according to relevant guidelines and regulations. Animals were housed in standard, single ventilated cages with 12 h light–12 h dark cycle and had ad libitum access to water and chow. The house temperature was maintained as 20 ± 2  °C and the relative humidity was kept as 50 ± 5%. All methods used are compliant with ARRIVE guidelines.

### Human tissue

Kidney samples were obtained from patients undergoing nephrectomies. Only histologically healthy parts of the kidney poles were used.

### Transgenic mouse lines

We generated a floxed NPR3mouse line (C57Bl/6J background, NPR3^fl/fl^; Cyagen) in which exon 3 was targeted. The line was crossed with podocin cre (B6.Cg-Tg [NPHS2-cre] 295Lbh/J; Jackson Laboratory) to generate a cell-specific knockout mice (NPR3^PodKO^). The Gt (ROSA)26Sor^tm14(CAG-td-Tomato)Hze/J^ mice were crossed with the podocin-cre line to activate the td-Tomato expression specifically in podocytes. The genotyping was done by PCR using genomic DNA extracted from ear biopsies. Primers for genotyping were: *NPR3*-LoxP-F: 5′ggtggcagaagatattttagggtttg3′, *NPR3*-LoxP-R: 5′cttaccccaggctgagcttcttt3′, *Cre*-F: 5′gcggtctggcagtaaaaactatc3′, *Cre*-R: 5gtgaaacagcattgctgtcactt5´.

### Anti-GBM glomerulonephritis model

NPR3^PodKO^ and C57Bl/6J control mic (9–11 weeks of age) were pre-immunized subcutaneously with 400µl/kg of sheep IgG Freund’s complete adjuvant (F5881, Sigma-Aldrich). Four days later, glomerulonephritis was induced by an intravenous injection of 130 μl of nephrotoxic serum (NTS) purchased from Probetex (PTX-001S). Urine was collected (day 0, 7, 14) and animals were sacrificed, and organs collected 14 days after the induction of the disease. Histopathology of animals was evaluated by scoring at least 30 glomeruli/mice as “normal” or “abnormal” (defined by presence of mesangial expansion, segmental sclerosis, crescents, and necrosis). Albuminuria was analyzed by running 2 µl of urine on SDS-PAGE gel (Invitrogen) and stained with SimplyBlue Safe stain (Invitrogen).

### Treatment of glomerulonephritis model with NPR3 inhibitor

NPR3 selective inhibitor (NPR3i; AZ12107657/M372049^15^) (15mg/kg) or vehicle (water/DMSO 50/50) was delivered continuously throughout the treatment period via Alzet micro-osmotic pumps (model 1002, Agnthos AB, Sweden). The osmotic pumps were implanted subcutaneously one day prior to the induction of glomerulonephritis in mice according to manufacturer’s instructions. The mice were followed up with body weight and urine collected every other day. On the final day mice were anesthetized with isoflurane followed by cervical dislocation to proceed with urine, blood, and organ collection.

### Rat diabetic kidney injury model

Male ZSF1 rats were purchased at 10-week of age from Charles River (Charles River, USA). Two groups of obese (ZSF1-*Lepr*^*fa*^*Lepr*^*cp*^/Crl 378), and lean (ZSF1-*Lepr*^*fa*^*Lepr*^*cp*^/Crl 379) animals were used in the following experiments. A total of 8-lean animals were used as a control group throughout the experimental setup. After 2 weeks of acclimation, obese ZSF1 rats were uninephrectomised (UNx) by removal of the right kidney. The animals underwent a two-week recovery period, followed by randomisation into different treatment groups (n = 10/treatment group). Treatment groups included: lean = unchallenged lean rats; vehicle = ZSF1, UNx rats treated with subcutaneous injection of a vehicle; NPR3i = ZSF1, UNx rats treated with subcutaneous injection with NPR3i; ARB = ZSF1, UNX rats treated with Losartan in drinking water, NPR3i + ARB = ZSF1, UNx rates treated with subcutaneous injection with NPR3 selective inhibitor and Losartan in drinking water. For dosing of the different treatments, the following was used: vehicle: normal saline (pH 5–6) (1 ml/kg), three times a week subcutaneous injection (week 1–5) then once daily subcutaneous injection (week 6–10). NPR3i: NPR3i (15 mg/kg, 1 ml/kg), three times a week subcutaneous injection (week 1–5), once daily subcutaneous injection week (6–10). Losartan: Losartan potassium (PHR1602-1G, Sigma-Aldrich) (20 mg/kg/day), in drinking water.

The animals were kept for a total of 10 weeks under treatment with constant follow-up and monitoring. Body weight and blood glucose were measured at baseline and once a week after the initiation of treatment. Animals were placed in metabolic cages at baseline, after 4 and 9 weeks of treatment for urine collection. After 10-weeks rats were anesthetized with isoflurane followed by exsanguination and disruption of blood flow.

### Quantitative PCR

Glomeruli were isolated from human and mouse kidneys as published previously^16^. Mouse podocytes from tdTomato mice were isolated as described^16^. Total RNA was extracted using RNeasy mini kit (Qiagen). First-strand cDNA synthesis was carried out using the iScript cDNA Synthesis Kit (BioRad). For real-time qPCR analysis, the CFX96Real-Time PCR Detection System and iQ SYBR Green Supermix (Bio-Rad) were used. The relative gene expression was calculated with the 2^−ΔΔCq^ method normalizing the gene of interest to housekeeping gene in the same sample. Data are presented as relative fold-change. Used primers can be found in supplemental Table 1.

### Immunostainings

Human frozen kidney sections were fixed in ice-cold acetone and blocked using 5% normal goat serum solution for 1 h at room temperature. Mouse kidneys were fixed with 4% paraformaldehyde and subsequently embedded with paraffin. Paraffin-embedded kidney sections (4 μm) were processed with antibodies to the antigens described below. For immunofluorescent studies, after HIER treatment with Tris–EDTA pH 9 for 20 min at 100 °C, sections were blocked using 5% normal goat serum solution for 1 h at room temperature.

Frozen and/or paraffin sections were incubated with NPR3 (ab97389, abcam, 1:1000), Synaptopodin (61094; Progen, 1:700), CD31 (303105; Biolegend, 1:500), PDGFRβ (MAB1263; R&D System, 1:1000), Nephrin (BP5030; Origene, 1:200), Wilms-Tumor1 (WT1) (ab89901; abcam, 1:100), ⍺-smooth muscle actin (A5228; Merck, 1:1000), tdTomato (TA150128; Origene, 1:50) at 4 °C overnight. Alexa Fluor conjugated secondary antibodies (Invitrogen) were used for visualization. All slides were co-stained with Hoechst 33342 (H3570, Life Technologies: 1:10,000). For paraffin embedded sections an extra step of incubation with sudan black B (0.1% in 70% ethanol, #199664, Sigma-Aldrich) solution was applied before mounting in Dako fluorescent medium (S3023, Agilent).

### Transmission electron microscopy

Samples for TEM were fixed in 2.5% glutaraldehyde and processed by the electron microscopy unit core facility (EMil) at Karolinska Institutet, Huddinge University hospital following standard procedures. Electron microscopic exmanitaion was performed by FEI Tecnai Spirit BioTWIN.

### Sanger sequencing

cDNA from wildtype (WT) and conditional knockout mouse glomeruli (NPR3^PodKO^) was amplified using PCR. The amplification product was isolated using Zymoclean Gel DNA Recovery Kit (ZymoResearch) according to the manufacturer’s instructions. Sanger sequencing was performed at KIGene facility at Karolinska Institutet, Solna following standard protocols.

### Biochemistry

Serum and urine cGMP were measured using enzyme immunoassay kit (#CG201-1KT, Sigma-Aldrich). Creatinine was measured using Quantichrome creatinine assay kit (#DICT-500, Bioassay systems) respectively. The urinary albumin/creatinine ratio was measured by using the commercial kits Albuwell (#1011, Exocell).

### Statistical analysis

Statistical analysis was performed with GraphPad Prism software (La Jolla, CA). For in vivo*, *in vitro and ex vivo experiments, data were analyzed using Student’s *t*-test or one-way ANOVA followed by Tukey’s multiple comparisons test when appropriate. For multiple treatment groups comparisons in the diabetic rat model mixed model ANOVA was used. A *p* value inferior to 0.05 was considered statistically significant. All data are presented as mean ± SD.

## Results

### NPR3 is highly expressed by mouse and human podocytes

**Figure 1:**
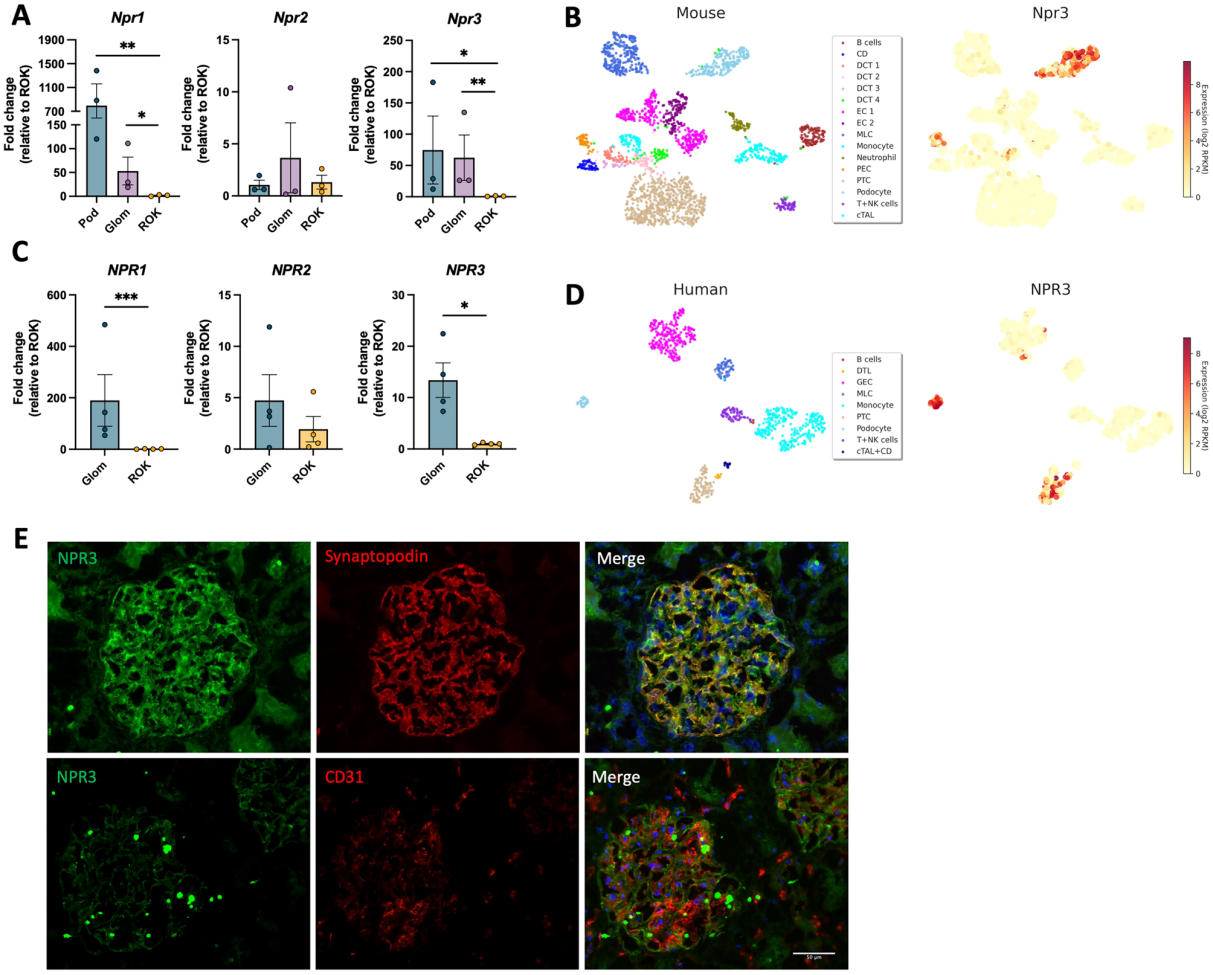
Expression of NPR3 in normal human and mouse kidneys. (**A**) RT-qPCR for NPR3 in FAC-sorted mouse podocyte (Pod), glomerular (Glom) and rest of kidney fractions (ROK). GAPDH was used as a house keeping gene. (**B**) Mouse single-cell RNA-sequencing data extracted from Kidney glomerular single cell atlas (data available at https://patrakkalab.se/kidney/). (**C**) RT-qPCR for NPR3 in human glomerular (Glom) and rest of kidney fractions (ROK). 28S was used as a housekeeping gene. (**D**) Human single-cell RNA-sequencing data extracted from Kidney glomerular single cell atlas (data available at https://patrakkalab.se/kidney/). The left panels show a t-SNE plot of cell clusters based on the specific cell markers. The right panels show t-SNE plot of NPR3 expression in the different clusters. *CD* collecting ducts, *DCT* distal convoluted tubules, *EC* endothelial cells, *MLC* mesangial-like cells, *PEC* glomerular parietal epithelial cells, *PTC* proximal tubular cells, *T + NK* T + natural killer lymphocytes, *cTAL* cortical thick ascending limb of Henle’s loop, *DTL* descending thin limb of Henle’s loop, *GEC* glomerular endothelial cells, *cTAL + CD* cortical thick ascending limb of Henle’s loop + collecting duct. (**E**) Immunofluorescence labelling in human kidney glomeruli for NPR3 (green) with podocyte marker Synaptopodin (red) and endothelial cell marker CD31 (red). Values are expressed as mean ± SD (n = 3). *p ≤ 0.05; **p ≤ 0.01; ***p ≤ 0.001.

The expression pattern of NPRs was assessed in murine and human kidney tissue. Quantitative-PCR analysis showed a significantly higher expression of NPR1 and NPR3 in the glomerular tissue compared to the tubulointerstitial fraction in mouse, and FACS-sorted mouse podocytes demonstrated highest NPR3 expression (Fig. 1A). Analysis of our in-house glomerular scRNA-seq data^17^ confirmed robust and predominant NPR3 expression in mouse podocytes with much lower expression detectable in mesangial and glomerular parietal epithelial cells (Fig. 1B) (supplemental Fig. 1).

In human kidney tissue, qPCR showed a similar enrichment of NPR1 and NPR3 in glomerular tissues as observed in mouse (Fig. 1C). The analysis of human glomeruli scRNA-seq data demonstrated NPR3 expression predominantly restricted to podocytes among the glomerular cell types (Fig. 1D). NPR3 expression was also noted in tubular epithelial cells (Fig. 1D and supplemental Fig. 1). Immunofluorescence staining of human kidneys validated podocyte NPR3 expression as shown by co-localization with podocyte marker synaptopodin, and weak signal was also detected in proximal tubular cells (Fig. 1E). Negative controls in which primary antibodies was omitted showed no significant staining (supplemental Fig. 2). Of note, despite our efforts, no reliable NPR3 antibody was validated for immunolocalization using mouse tissues (data not shown).

### NPR3 inactivation in podocytes does not result in glomerular abnormalities or modulate the outcome of nephropathy in glomerulonephritis model

**Figure 2:**
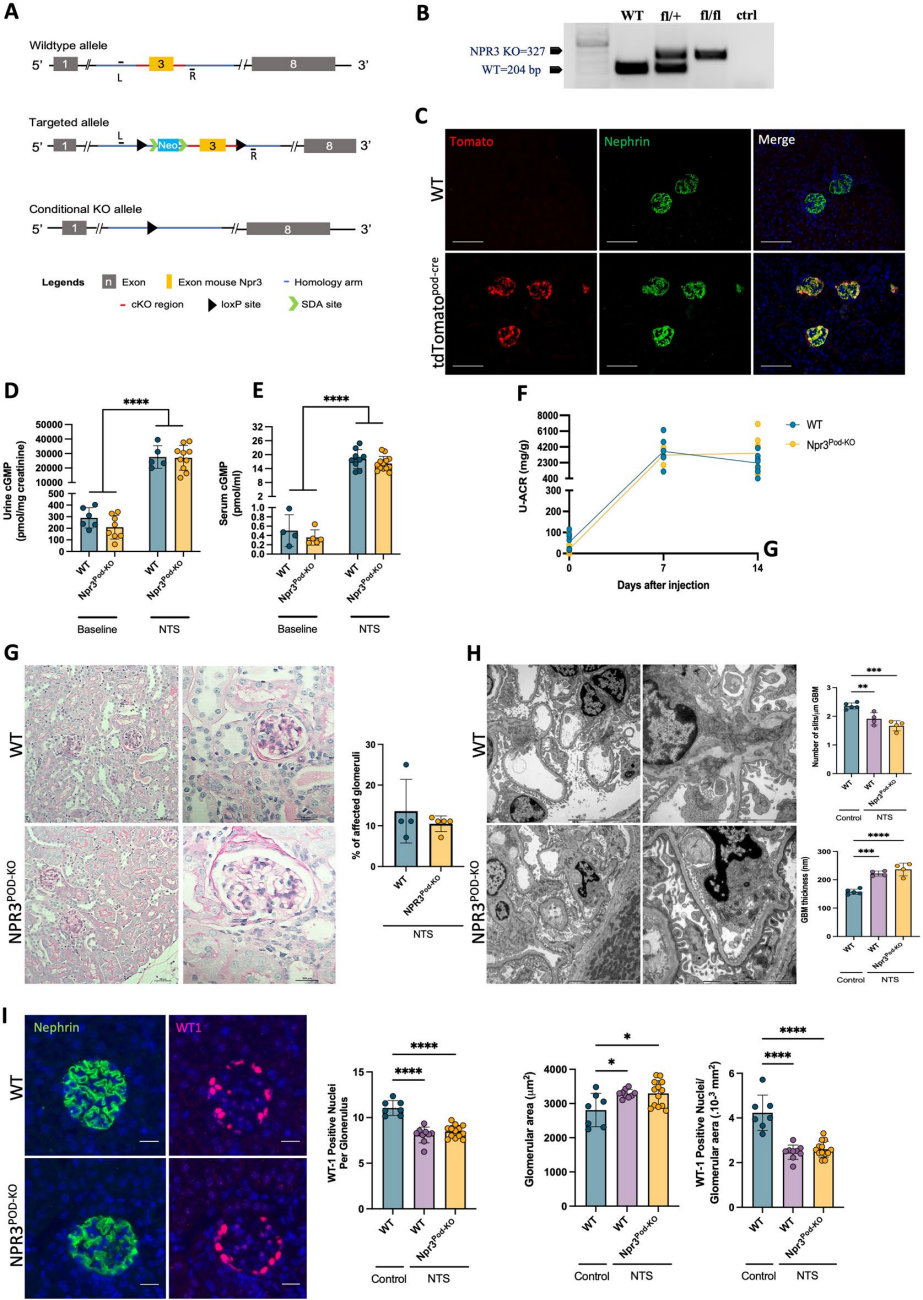
Characterization of NPR3 ^Pod-KO^ at baseline and after NTS-induced injury. (**A**) Generation of conditional knockout mice in which NPR3 is specifically ablated in podocytes using Cre–LoxP recombination system. Exon 3 is deleted upon NPHS2-Cre-mediated recombination (L/R = left/right genotyping primer). (**B**) Genotyping by ear preparation and PCR at 4 weeks of age. (**C**) Expression of Tomato-reporter gene (red) and Podocyte marker Nephrin (green), scale bar; 100 μm. (**D**) Urinary cGMP levels in WT and NPR3^Pod-KO^ mice, before and after NTS challenge (urine cGMP WT; n = 6, urine cGMP NPR3^Pod-KO^; n = 8, urine cGMP NTS WT; n = 5, urine cGMP NTS NPR3^Pod-KO^; n = 10). (**E**) Serum cGMP levels in WT and NPR3^Pod-KO^ mice, before and after NTS challenge (serum cGMP WT; n = 4, serum cGMP NPR3^Pod-KO^; n = 5, serum cGMP NTS WT; n = 9, serum cGMP NTS NPR3^Pod-KO^; n = 13). (**F**) Urinary albumin/ creatinine ratios (U-ACR) in WT and NPR3^Pod-KO^ after NTS-induced glomerulonephritis (WT; n = 5, NPR3^Pod-KO^; n = 6). (**G**) Histology and quantification of affected glomeruli in NTS-challenged mice. 30 randomly selected glomeruli were evaluated using PAS staining (WT; n = 4, NPR3^Pod-KO^; n = 5). (**H**) Electron microscopic findings and quantification of mean glomerular basement membrane (GBM) thickness and the number of slits per μm GBM in control and NTS challenged mice. Quantification was performed from transmission electron microscopy images (control; n = 5, WT; n = 4, NPR3^Pod-KO^; n = 4). (**I**) WT1 staining and quantification of positive cells in glomeruli, glomerular area and WT1 positive podocytes/glomerular area control and NTS challenged mice. Nephrin staining (green) was used for the quantification of glomerular area and WT1 (red) for number of podocytes. Quantification was performed from immunofluorescent images (control; n = 7, WT; n = 9, NPR3^Pod-KO^; n = 13). *p ≤ 0.05; **p ≤ 0.01; ***p ≤ 0.001, ****p ≤ 0.0001.

To investigate the role of NPR3 in glomerular function, we generated a podocyte-specific knockout mouse line (NPR3^PodKO^). We produced a novel mouse line in which exon 3 of the mouse NPR3 gene was floxed and crossed it with a podocin-cre mouse line (Fig. 2A). Ear genotyping showed the NPR3 wildtype band at 204bp and knockout band at 327bp (Fig. 2B). As we did not have a reliable antibody to measure NPR3 expression in mouse tissue, we used PCR to demonstrate the deletion of exon 3 specifically in the glomerulus (supplemental Fig. 3a). The deletion of exon 3 in a knockout band was validated by Sanger sequencing (supplemental Fig. 3b). As we detected also a wildtype band, we showed the efficacy of the podocin-cre line by crossing it with a tdtomato line. The expression of Tomato-reporter gene was detected in nephrin-positive podocytes (Fig. 2C). When quantified the expression of Tomato-reporter gene expression was consistently detected in > 95% of nephrin-positive podocytes (data not shown). Thus, we speculate that the remaining wild-type product represented NPR3 expression originating from other cell types.

NPR3^PodKO^ animals were viable, fertile, and developed normally. Histological analysis of the kidneys showed no overt differences when compared to their littermate controls (supplemental Fig. 3c) and no albuminuria was detected when followed up to 12 months of age (supplemental Fig. 3d). Since NPR3 acts as a clearance receptor for NPs both systematically and locally^3,17^, the urinary and systemic cGMP levels were assessed as a potential indicator of increased natriuretic peptide signaling at NPR1/2. No significant differences were observed between the WT and NPR3^PodKO^ mice (Fig. 2D, E).

To elucidate the possible involvement of NPR3 in the development of glomerular damage, we first analyzed how the absence of NPR3 affects the progression of acute glomerular injury. For this, we induced glomerulonephritis in mice with a single i.v. injection of NTS as previously described^18^. Mice developed albuminuria that peaked at day 7, however, no differences between NPR3^PodKO^ and WT mice were seen over the time course examined (Fig. 2F). Histopathological evaluation of kidneys collected at day 14 showed no significant differences in the level of glomerular injury achieved based on NPR3 genotype (Fig. 2G). Electron microscopic evaluation demonstrated similar injury-induced characteristic ultrastructural changes to the glomerular filtration barrier, with no quantifiable differences in the thickness of the GBM or foot process effacement (Fig. 2H). Furthermore, the evaluation of glomerular hypertrophy and WT1 positive podocytes showed no significant differences between NPR3^PodKO^ and WT mice (Fig. 2I). Of note, an increased level of cGMP in both urine and serum was seen in NTS mice vs. baseline (Fig. 2D, E).

### Pharmacological inhibition of NPR3 ameliorates podocyte loss and glomerular damage in glomerulonephritis model

**Figure 3:**
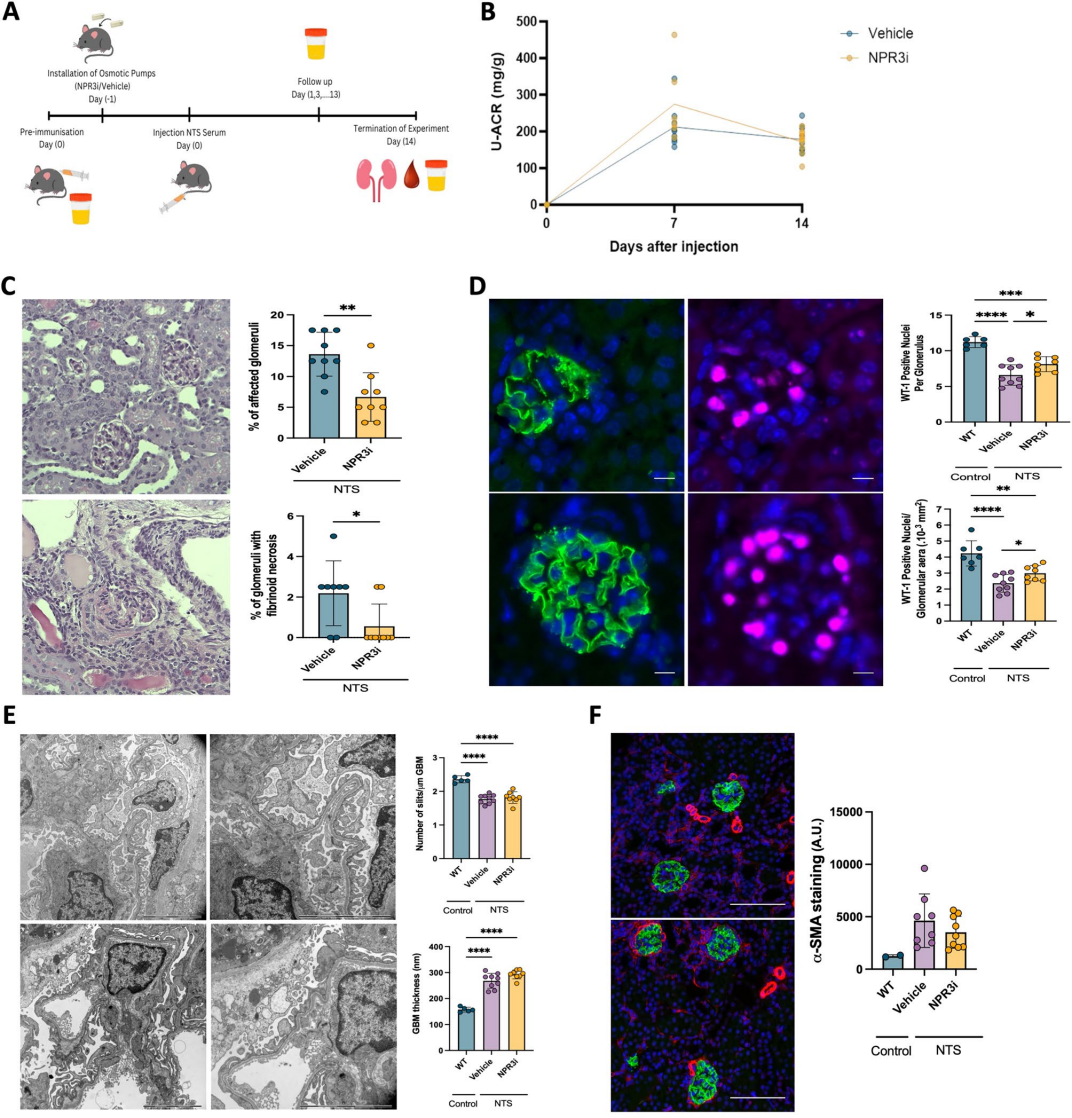
Treatment with NPR3 inhibitor via subcutaneous osmotic-pumps in mice with NTS-induced glomerular injury. (**A**) Schematic representation of the experimental treatment setup. (**B**) Urinary albumin/ creatinine ratios (U-ACR) in vehicle and NPR3^i-treated^ mice after induction of glomerulonephritis (n = 5 for both). (**C**) Histology and quantification of affected glomeruli in treated mice. 30 randomly selected glomeruli were evaluated using PAS and Masson Trichrome staining (vehicle; n = 8, NPR3i; n = 9). (**D**) WT1 staining and quantification of positive cells in glomeruli, glomerular area and WT-1 positive podocytes/glomerular area in control and treated mice. Nephrin staining (green) was used for the quantification of glomerular area and WT1 (red) for number of podocytes. Quantification was performed from immunofluorescent images; 20 randomly selected glomeruli were evaluated (WT; n = 6, vehicle; n = 9, NPR3i; n = 8). (**E**) Electron microscopic findings and quantification of mean glomerular basement membrane (GBM) thickness and the number of slits per μm GBM in control and NTS challenged mice. Quantification was performed from transmission electron microscopy images (control; n = 5, WT; n = 4, NPR3^Pod-KO^; n = 4). (**F**) Staining and quantification of fibrosis marker ⍺-sma positive area in kidney cortex of control and treated mice. Quantification was performed from immunofluorescent images; an average of 10–12 randomly selected cortical cross sections with visible glomeruli were evaluated (WT; n = 2, vehicle; n = 8, NPR3i; n = 9). Scale bars: 100 μm. *p ≤ 0.05; **p ≤ 0.01; ***p ≤ 0.001, ****p ≤ 0.0001.

Next, we wanted to analyze whether pharmacological inhibition of NPR3 modulated the outcome of the NTS-induced glomerular injury. We administrated a selective NPR3 blocking peptide (NPR3i)^15^ using subcutaneous osmotic pumps and followed up mice for 14 days (Fig. 3A). No difference was detected in the levels of albuminuria at day 7 or day 14 (Fig. 3B). NTS-challenged mice treated with NPR3i showed less histological changes and fibrinoid necrosis in glomeruli when compared to vehicle treated littermates (Fig. 3C). Moreover, the number of WT1 positive cells per glomerulus was higher in NPR3i treated group suggesting that podocyte were partially protected (Fig. 3D). No difference was observed in GBM thickness or foot process effacement between the groups (Fig. 3E). The measurement of fibrosis in kidney cortex showed no significant change in ⍺-smooth muscle actin levels in NPR3i-treated animals (Fig. 3F). Of note, no compensatory expressional changes were detected for NPR1 and NPR2 in NPR3i-treated glomeruli (supplemental Fig. 4).

### Pharmacologic inhibition of NPR3 potentiates the protective effect of angiotensin receptor blocker (ARB) treatment in diabetic rat model

**Figure 4:**
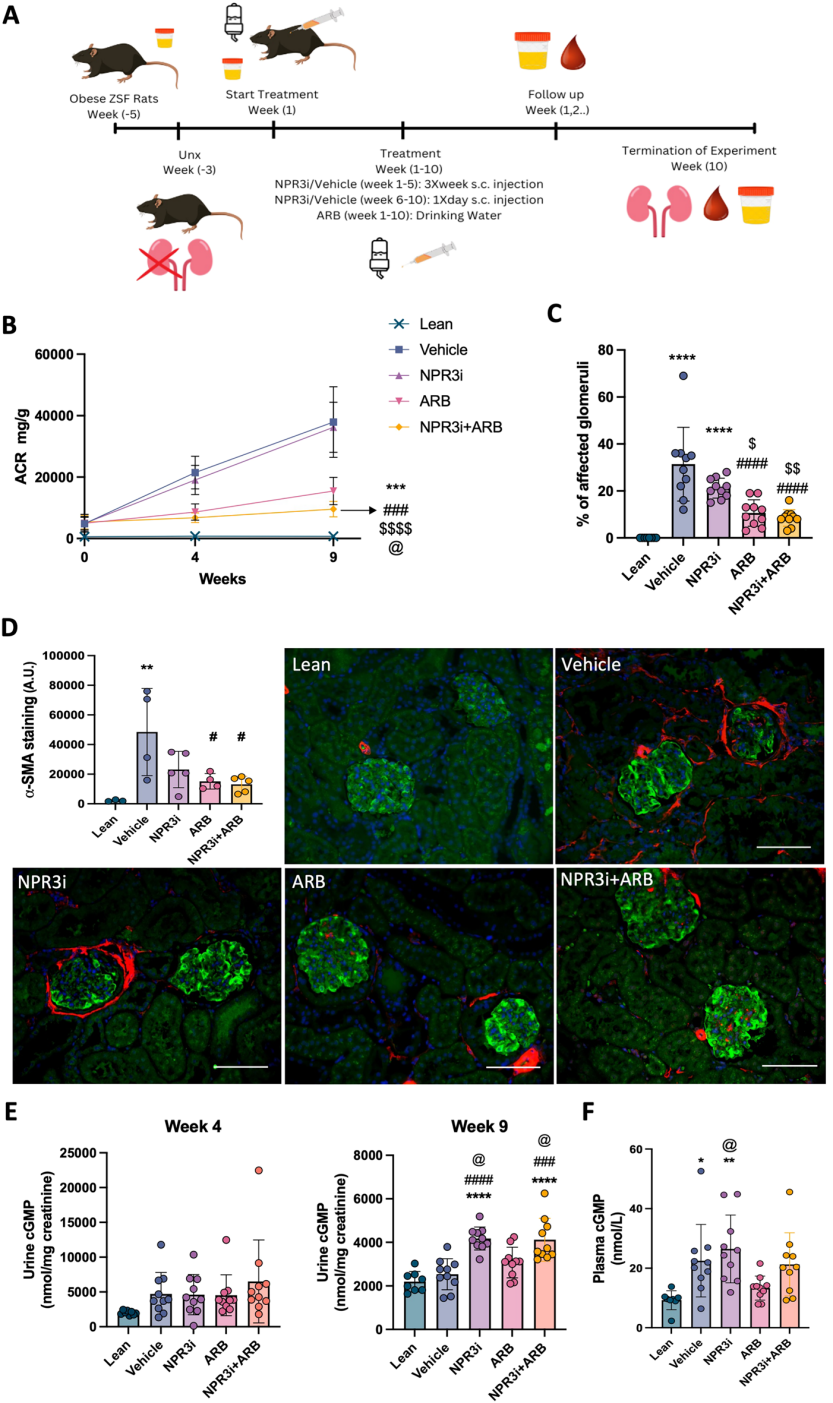
Treatment with selective NPR3 inhibitor monotherapy or combination therapy with ARB in diabetic nephropathy rat model. (**A**) Schematic representation of the experimental and treatment setup. (**B**) Urinary albumin/ creatinine ratios (U-ACR) in different treatment groups of diabetic nephropathy rat model (lean; n = 8, vehicle; n = 10, NPR3i; n = 10, ARB; n = 10, NPR3i + ARB; n = 10). (**C**) Quantification of affected glomeruli in treated rats. 30 randomly selected glomeruli were evaluated using PAS staining (lean; n = 8, vehicle; n = 10, NPR3i; n = 10, ARB; n = 10, NPR3i + ARB; n = 10). (**D**) Immunofluorescence and quantification of fibrosis marker ⍺-sma positive area in kidney cortex of control and treated mice (lean; n = 3, vehicle; n = 4, NPR3i; n = 5, ARB; n = 4, NPR3i + ARB; n = 5). Quantification was performed from immunofluorescent images; an average of 10–12 randomly selected cortical cross sections with visible glomeruli were evaluated. Nephrin staining (green) was used to locate the glomeruli and cortex area. ⍺-sma positive area (red) was used for fibrosis quantification. (**E**) Urinary cGMP levels at week 4 and 9 respectively corrected to creatinine levels in different treatment groups (lean; n = 8, vehicle; n = 10, NPR3i; n = 10, ARB; n = 10, NPR3i + ARB; n = 10). (**F**) Plasma cGMP levels in different treatment groups (lean; n = 8, vehicle; n = 10, NPR3i; n = 10, ARB; n = 10, NPR3i + ARB; n = 10). Treatment groups: lean = unchallenged lean rats, vehicle = ZSF1, UNx rats treated with subcutaneous injection of a vehicle (1 ml/kg), NPR3i = ZSF1, UNx rats treated with subcutaneous injection with NPR3 selective inhibitor (15 mg/kg, 1 ml/kg), ARB = ZSF1, UNX rats treated with Losartan in drinking water (20 mg/kg/day), NPR3i + ARB = ZSF1, UNx rates treated with subcutaneous injection with NPR3 selective inhibitor (15 mg/kg, 1 ml/kg) and Losartan in drinking water (20 mg/kg/day). Plasma cGMP levels in different treatment groups (lean; n = 8, vehicle; n = 10, NPR3i; n = 10, ARB; n = 10, NPR3i + ARB; n = 10). *p ≤ 0.05; **p ≤ 0.01; ***p ≤ 0.001, ****p ≤ 0.0001. *Compared to lean, #compared to vehicle, @compared to ARB, $compared to NPR3i. Scale bars: 100 μm.

To test whether NPR3 inhibition could have beneficial effects in a chronic model of glomerular damage, we treated nephrectomised obese ZSF1 rats (UNx-ZSF1) with NPR3i. As a combination of neprilysin inhibition (targeting NP signaling) with ARB has previously shown protective effects in DN ^10,19^, we compared NPR3i monotherapy to not only vehicle-treated but also NPR3i and ARB combination therapy in UNx-ZSF1 rat model (Fig. 4A).

UNx-ZSF1 rats presented with a progressive increase of albuminuria over the duration of treatment and glomerulosclerosis at termination (Fig. 4B, C). Treatment with NPR3i alone did not impact the temporal increase in albuminuria levels (Fig. 4B). ARB treatment significantly reduced albuminuria and notably, a significant further reduction in albuminuria was seen in NPR3i + ARB combination group when compared to ARB alone (Fig. 4B). In the histological examination, there was no significant reduction in glomerular damage NPR3i alone group as observed by histological scoring (p = 0.051) (Fig. 4C). ARB treatment resulted, on the other hand, in reduced glomerular histopathological changes (Fig. 4B). NPR3i + ARB group showed slightly less changes in comparison to ARB-treated animals (Fig. 4B). This was not, however, significant. No therapeutic differences were observed in GBM thickness, foot process effacement, glomerular tuft area, or podocyte loss, when comparing different treated groups to the vehicle (supplemental Fig. 4). Fibrosis in kidney cortex was significantly reduced with ARB and with NPR3i + ARB treatment when compared to the vehicle treated group (Fig. 4D). Of note, NPR3i increased urinary ANP levels (supplemental Fig. 4c), while plasma ANP only modestly increased in NPR3i + ARB group (supplemental Fig. 4d).

Urinary cGMP levels, which we used as a marker for increased NP-mediated glomerular signaling, showed a significant increase in both NPR3i and NPR3i + ARB treatment groups after 9 weeks of treatment with NPR3i (Fig. 4E). Plasma cGMP levels were increased in only NPR3i group (Fig. 4E). Of note, cGMP levels were not significantly altered 4 weeks after the start of therapy.

## Discussion

NPs are critical for the maintenance of cardiovascular and kidney homeostasis. The important role of NP-system in the glomerulus has been highlighted by a study showing that NPR1-deficiency in podocytes promotes glomerular injury in disease models^4,20^. We discovered a robust expression of clearance receptor NPR3 in podocytes. To address whether NPR3 had a role in the maintenance of glomerular homeostasis, we generated a podocyte-specific KO mouse line. These mice showed no renal abnormalities indicating that NPR3 was not needed for the maintenance of normal glomerulus function. Moreover, no significant differences were detected between NPR3^PodKO^ and control animals after the induction of glomerulonephritis. Thus, our data argues that podocyte-derived NPR3 does not have a significant role in the regulation of glomerular function. The lack of phenotypical differences in both healthy and diseased condition can be due to the wide distribution of NPR3 expression in different organs, which may compensate systemically for the lack of the receptor in podocytes^21,22^. Studies in inducible knockout model could be useful in exploring further this question.

To explore whether targeting NPR3 could be a therapeutical option for glomerulopathies, we treated an NTS-induced mouse glomerulonephritis model with NPR3-specific blocking peptide (NPR3i). NPR3i exerted reno-protective effects as shown by reduced histopathological changes and maintenance of WT1 positive podocytes in comparison to non-treated controls. This was clearly different from the effects seen in NPR3^PodKO^ animals. There can be several reasons for the discrepancy. We speculate primarily that the short-term blocking of the receptor using an inhibitor cannot be compensated systemically as in the case of KO animals. Moreover, it is plausible to think that some of the protective effects observed with NPR3i are mediated by extra-glomerular inhibition of NPR3.

To further examine these possibilities, we treated uni-nephrectomized ZSF1 rats with NPR3i. No significant protective effects were detected in this model with NPR3i treatment alone. This is similar to the previous studies using neprilysin inhibitors that also showed lack of efficacy in various rodent disease models^23^. However, as neprilysin inhibitors have been combined successfully with ARBs in the treatment of cardiovascular diseases and with promising prospects in diabetic nephropathy ^8–10,19^, we treated our rat model also with a combination of NPR3i and ARB. The combination therapy seemed to provide additional protection as shown by a significant decrease in albuminuria levels in comparison to losartan-treated group. However, no difference was detected in other read-outs of renal function or histology as the RAAS inhibitory effect of losartan is requisite and dominant in this model, making it challenging to capture additive or synergistic effects. The mechanism for potentiation of reno-protective effects of losartan is unclear. It can be that NPR3-inhibition and losartan have independent reno-protective effect. ARBs target mainly efferent arterioles and diminish glomerular filtration pressure, whereas NPR3-inhibition targets podocytes and potentially promotes directly podocyte health.

Our study among other recently published studies ^15,26^ demonstrate NPR3 as a potential target to manipulate NP signaling. Previous in vitro studies have shown the effectiveness of NPR3 blockage^13,21^, along with kidney protective results observed in vivo^13,26^. Nevertheless, the sensitive nature and rapid degradation of targeting peptides on one hand, and the tissue distribution of NPR3 and compensatory mechanisms on the other, imposes various challenges to developing a therapeutic NPR3 blocker for treating chronic diseases. Finding “the sweet spot” with the right dosage, drug delivery system and suitable animal models to test it is needed before further advancements with NPR3i as a therapeutic agent.

There are few limitations in this work that should be highlighted. First, we did not measure blood pressure in our animal experiments. In previous studies where NPR3 was targeted, no differences were seen on the blood pressure measurement in kidney disease models ^13,26^, additionally, no previous studies have shown an elevation of blood pressure in an NTS model. Yet we cannot exclude that some of the effects observed were mediated by changes in blood pressure. Second, we could not validate at the protein level the inactivation of NPR3 in our knockout model. However, RT-PCR and studies in a reporter mouse argue strongly for an effective NPR3 deletion in podocytes. A clear wildtype product in KO glomeruli could represent a limited NPR3 expression in other glomerular-intrinsic cell types ^24,25^ and non-glomerular nephron segments that contaminate an otherwise homogeneous glomerular preparation. Third, in our mouse model treated with NPR3i, we did not measure target engagement due to the relatively low compound exposure achieved with minipump administration and limited amounts of samples available. Additional inducible kidney injury models (e.g. streptozotocin-induced diabetes, adriamycin-induced podocyte injury) as well as podocyte-toxin targeted genetic injury models on a background of NPR3-podocyte deletion need to be explored and should help to further elucidate the protective effects of natriuretic peptides on podocyte health. Lastly, we cannot exclude that non-specific off-target effects are behind some of the findings in NPR3i-treated animals. The fact that KO animals do not show any renal protection supports this idea. However, this difference could be explained by the temporary nature of NPR3 inhibition under NPR3i-therapy.

In conclusion, this study suggests that pharmacologic blocking of NPR3 can be a possible strategy for the treatment of glomerular diseases. Further studies, including compound and dose optimization as well as finding the right route of delivery are needed to further interrogate and validate the therapeutic potential of NPR3 targeting to treat kidney disease.

### Supplementary Information

Supplementary Information.

## Data Availability

All data generated or anlysed during this study are included in this published article (and its supplementary information files).
