# Supplementary material for: Unraveling the role of natriuretic peptide clearance receptor (NPR3) in glomerular diseases
Source: Sci Rep. 2024 May 24;14:11850. doi: 10.1038/s41598-024-61603-4 (PMC11116399; doi:10.1038/s41598-024-61603-4)
Supplement: Supplementary file 1 — Supplementary Information. [file 41598_2024_61603_MOESM1_ESM.pdf]

Supplementary Figure 1. Expression of different natriuretic peptide receptors in two single-cell RNA-sequencing databases.

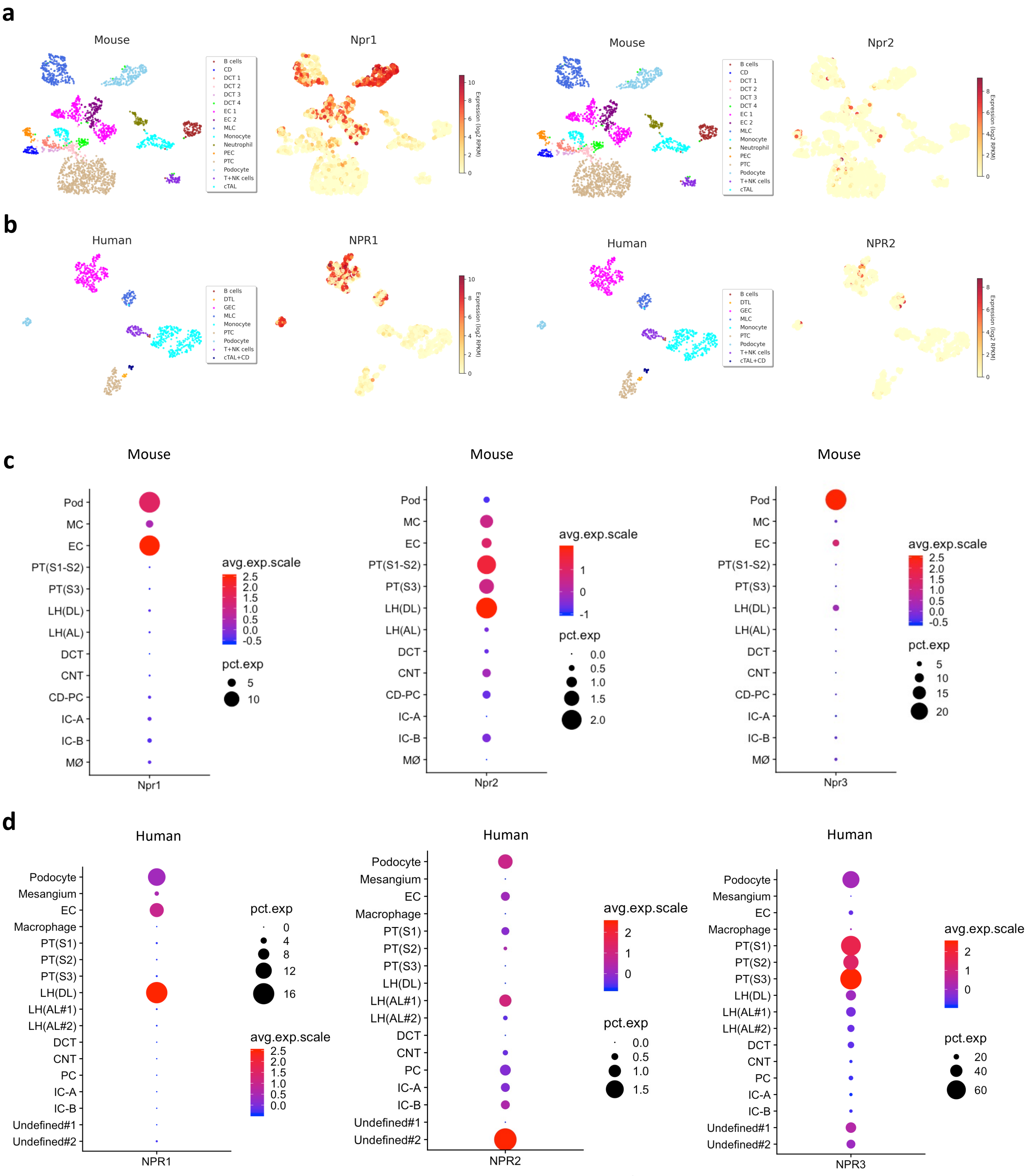

a. Human and mouse single-cell RNA-sequencing data extracted from Kidney glomerular single cell atlas (data available at https://patrakkalab.se/kidney/). The left panels show a t-SNE plot of cell clusters based on the identification of highly expressed specific cell markers. The right panels show t-SNE plot of NPR1 expression in the different clusters. CD: collecting ducts; DCT: distal convoluted tubules; EC: endothelial cells; MLC: Mesangial-like cells; PEC: glomerular parietal epithelial cells; PTC: Proximal tubular cells; T+NK: T + natural killer lymphocytes; cTAL: cortical thick ascending limb of Henle’s loop. DTL: descending thin limb of Henle’s loop; GEC: glomerular endothelial cells; cTAL + CD: cortical thick ascending limb of Henle’s loop + collecting duct. **b.** Human and mouse single-cell RNA-sequencing data extracted from Kidney glomerular single cell atlas (data available at https://patrakkalab.se/kidney/). The left panels show a t-SNE plot of cell clusters based on the identification of highly expressed specific cell markers. The right panels show t-SNE plot of NPR1 expression in the different clusters. CD: collecting ducts; DCT: distal convoluted tubules; EC: endothelial cells; MLC: Mesangial-like cells; PEC: glomerular parietal epithelial cells; PTC: Proximal tubular cells; T+NK: T + natural killer lymphocytes; cTAL: cortical thick ascending limb of Henle’s loop. DTL: descending thin limb of Henle’s loop; GEC: glomerular endothelial cells; cTAL + CD: cortical thick ascending limb of Henle’s loop + collecting duct.**c-d.** Mouse and human single-cell RNA-sequencing data (respectively) extracted from mouse and human single cell atlas (data available at http://humphreyslab.com/SingleCell/). The panels show plots of NPR1, NPR2, and NPR3 expression different clusters. Pod: podocytes; MC: Mesangial Cells; EC: Endothelial Cells; PT (S1-S2): Proximal tubuli S1, S2 segments: PT (S3): Proximal tubuli S3 segment; LH (DL): Loop of Henle descending limb; LH (AL) Loop of Henle ascending limb; IC-A: Intercalated cell type A; IC-B: Intercalated cell type B; MΦ: macrophage; DCT: Distal convoluted tubule; CNT: Connecting tubule; PC: Principal cells.

Supplemental figure 2. Negative control stainings in human and mouse kidneys.

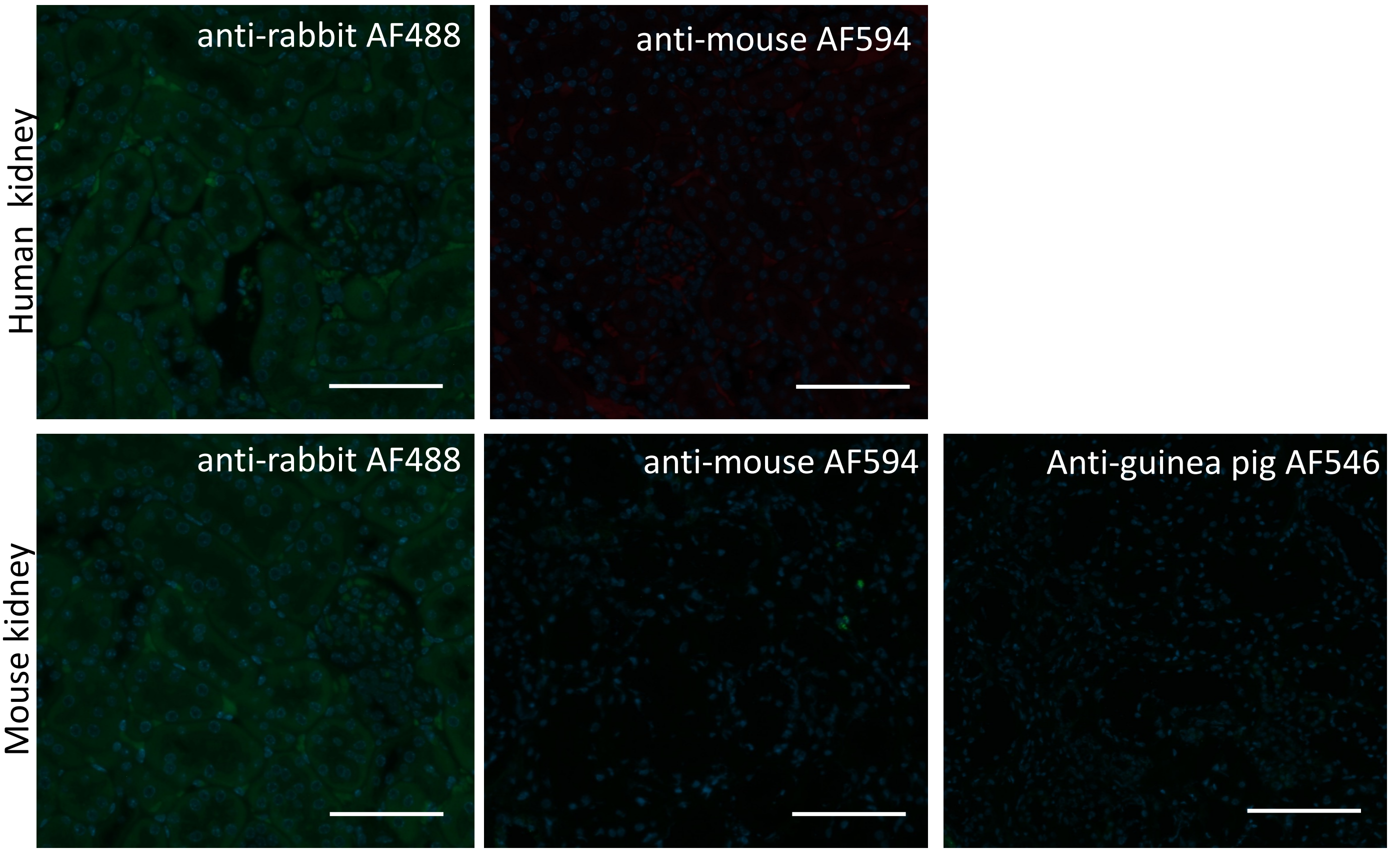

Human and mouse kidneys were processed as in other immunofluorescence experiments except primary antibody was omitted. Secondary antibodies (including fluorochromes) are indicated in images. Scale bars; 300 µm

Supplemental figure 3. Characterisation of NPR3$^{Pod\text{-}KO}$ mice.

a

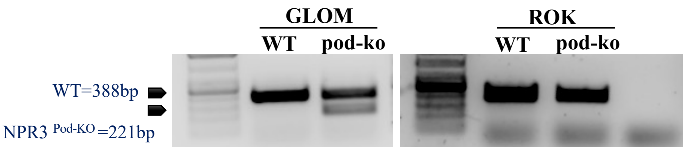

b

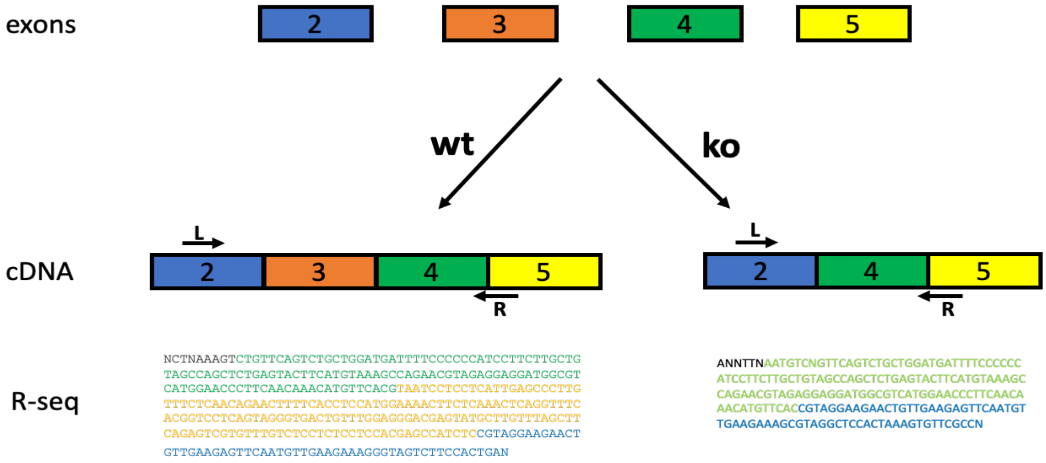

c

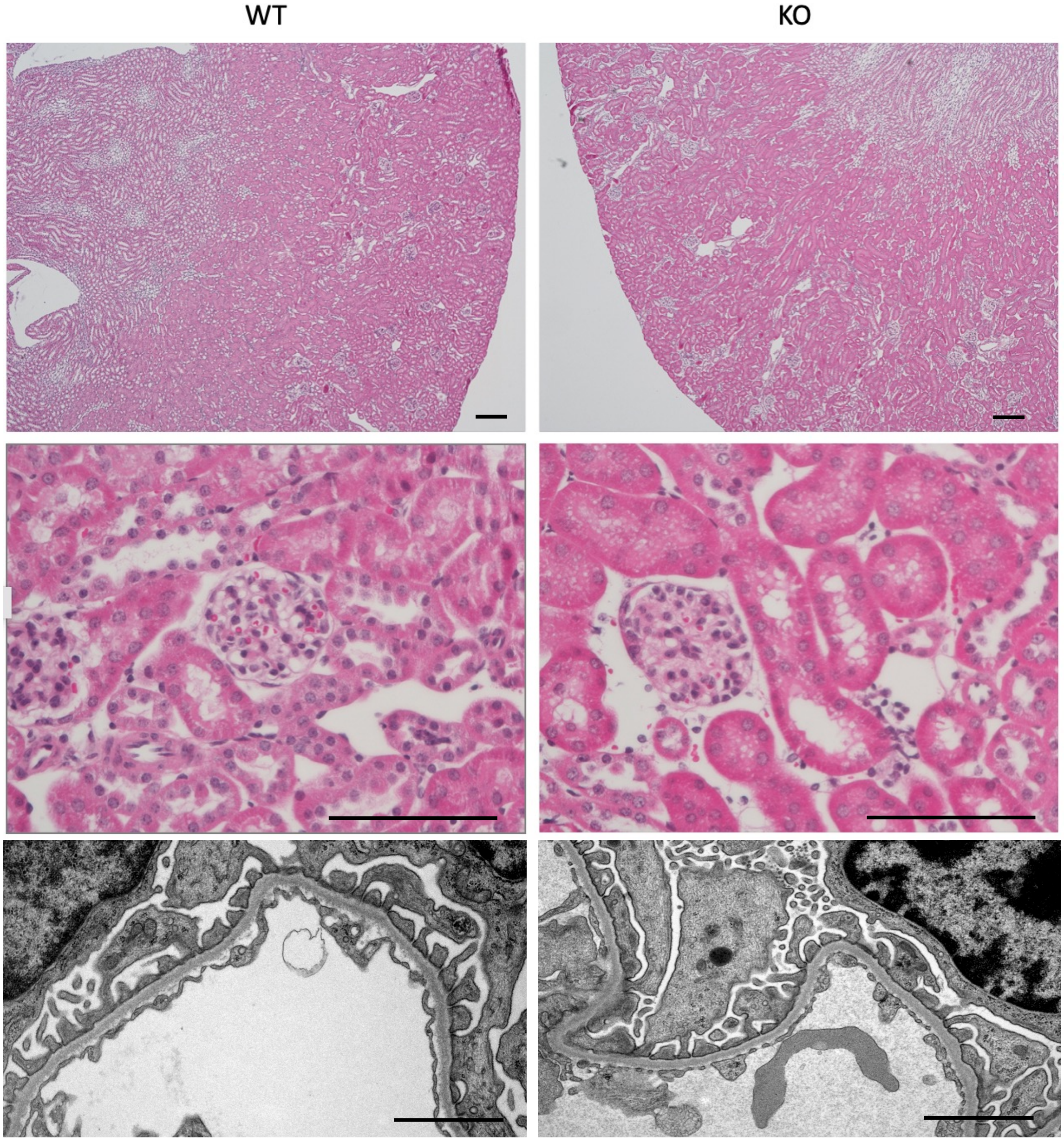

d

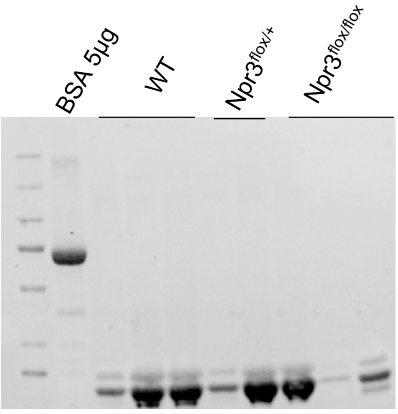

a. PCR amplification of cDNA from WT and NPR3$^{Pod\text{-}KO}$ isolated glomeruli using primers from exon 2 (forward) and exon 5 (reverse). Two PCR products observed: WT band at 388 bp and KO band at 221 bp. **b.** Sanger sequencing of the PCR products isolated from (B) using primers from exon 2 (forward) and exon 5 (reverse). In WT mice exon 2 (blue), exon 3 (orange), exon 4 (green) are detected. In NPR3$^{Pod\text{-}KO}$ mice only exon 2 (blue) and exon 4 (green) were detected**. c.** Histological comparison of wild type and knock-out kidneys in 10-week old mice. Bars in hematoxylin-eosin images; 300 µm. Bars in electron microscopy images; 1 µm **d.** Analysis of urine collected from WT and NPR$^{Pod\text{-}KO}$ animals in SDS PAGE gel.

Supplemental figure 4. Expression of NPR1 and NPR2 in NPR3i-treated mice.

a

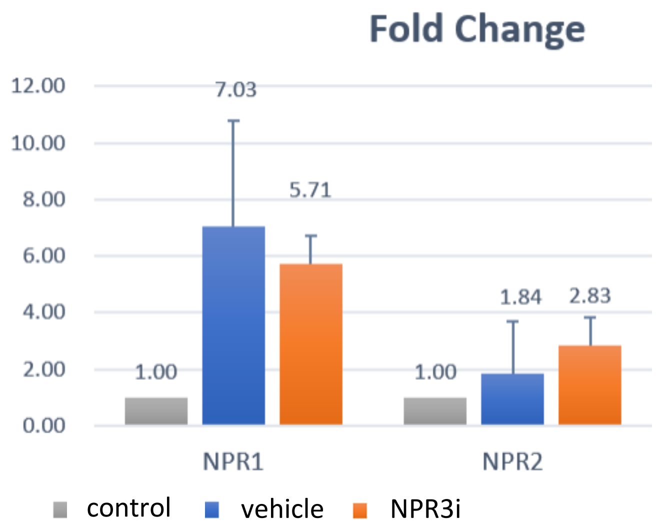

b

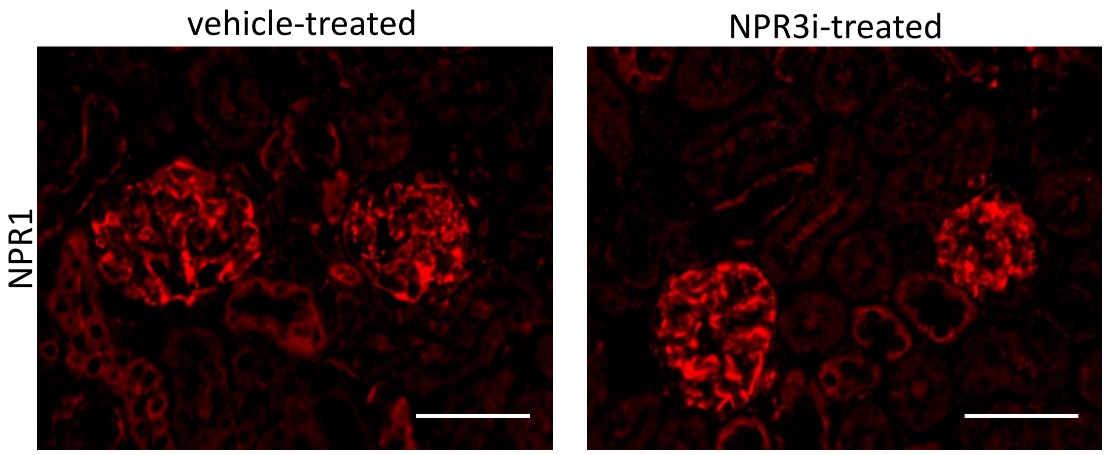

a. qPCR analysis of isolated glomerular fractions in vehicle and NPR3i-trated mice. **b.** Immunofluorescence for NPR1 in vehicle and NPR3i-trated mice. Bars; 300 μm

Supplemental figure 5. Therapeutic NPR3i in diabetic nephropathy (DN) model in rats.

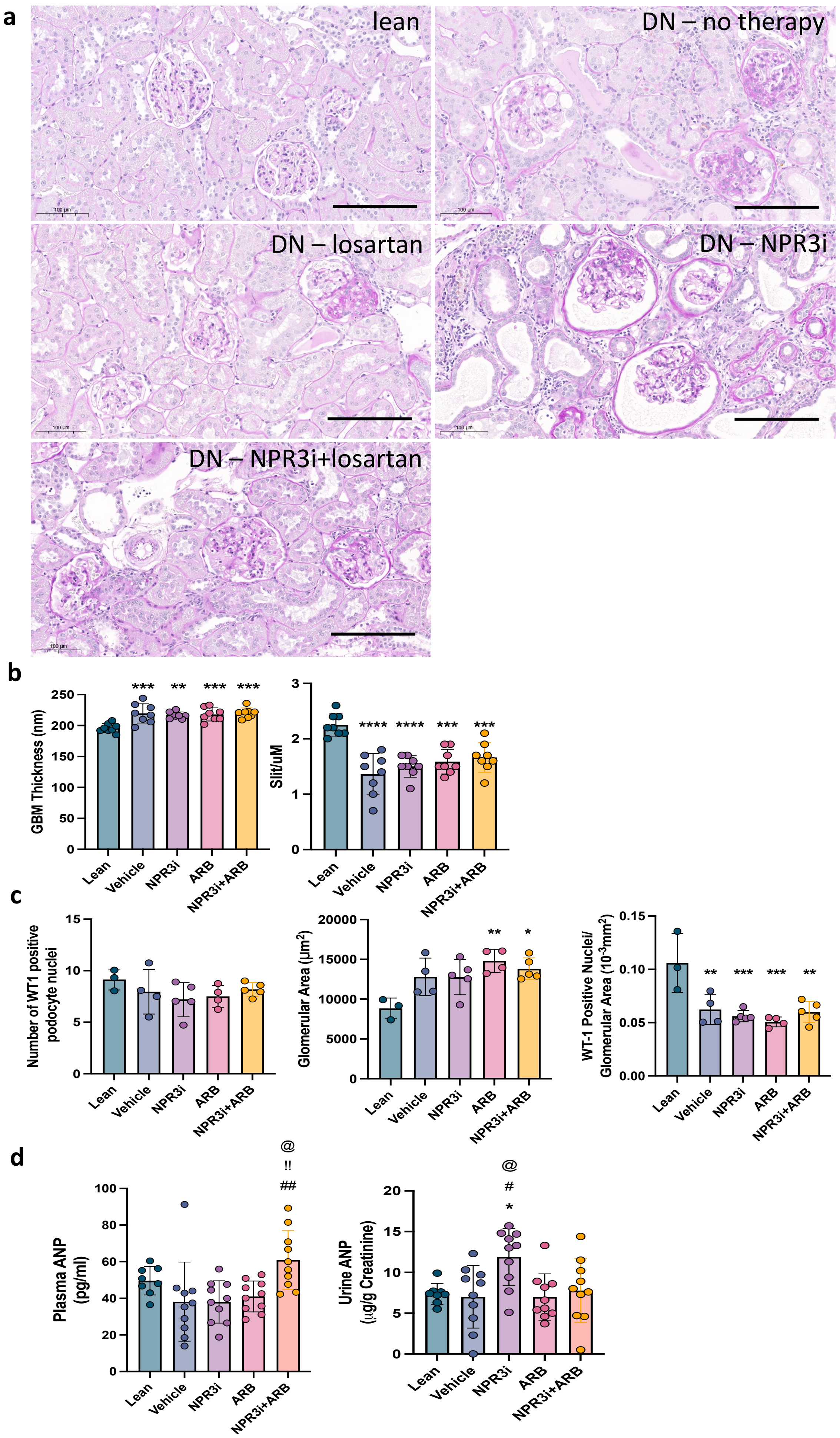

a. Histological findings in DN model under different therapies **b.** Quantification of mean glomerular basement membrane (GBM) thickness (left panel) and the number of slits per µm GBM (right panel) in control and treated mice. Quantification was performed from transmission electron microscopy images (Lean; n=8, Vehicle; n=8, NPR3i; n=8, ARB; n=8, NPR3i+ARB; n=8. **c.** Quantification of the number of WT-1 positive podocytes in glomeruli (left panel), glomerular area (middle panel) and WT-1 positive podocytes/glomerular area (right panel) in control and treated mice (Lean; n=3, Vehicle; n=4, NPR3i; n=5, ARB; n=4, NPR3i+ARB; n=5). Quantification was performed from immunofluorescent images; 20 randomly selected glomeruli were evaluated. **c.** Urinary Atrial natriuretic peptide (ANP) levels corrected to creatinine levels in different treatment groups (Lean; n=8, Vehicle; n=10, NPR3i; n=10, ARB; n=10, NPR3i+ARB; n=10). **d.** Plasma Atrial natriuretic peptide (ANP) levels in different treatment groups (Lean; n=8, Vehicle; n=10, NPR3i; n=10, ARB; n=10, NPR3i+ARB; n=10). * $p \le 0.05$; **$p \le 0.01$; *** $p \le 0.001$, **** $p \le 0.0001$. * Compared to Lean, # Compared to Vehicle, @ Compared to ARB, $ Compared to NPR3i.

Supplemental figure 5. Uncropped gels.

Figure 2b

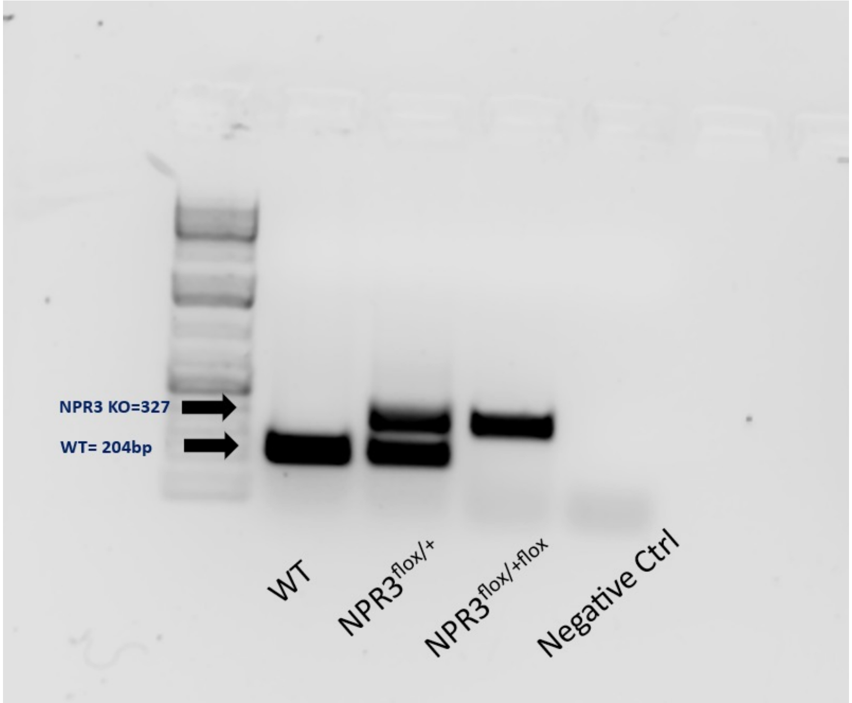

Supplemental figure 3a

Glom

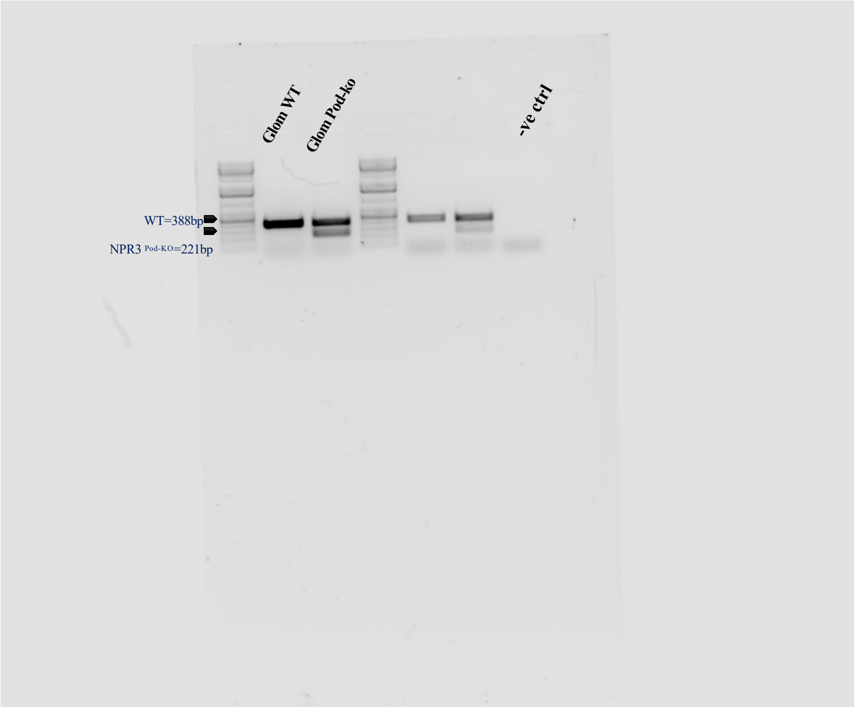

ROK

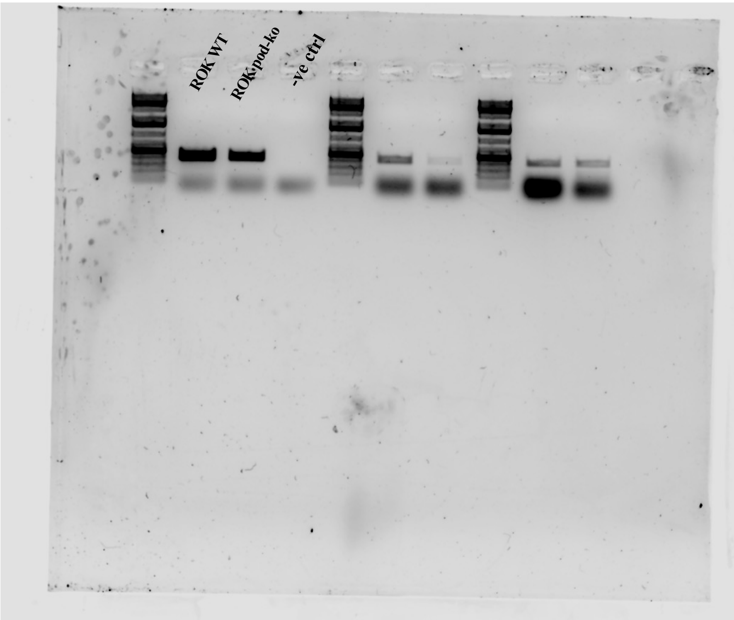

Supplemental figure 6. Uncropped gels.

Supplemental figure 3d

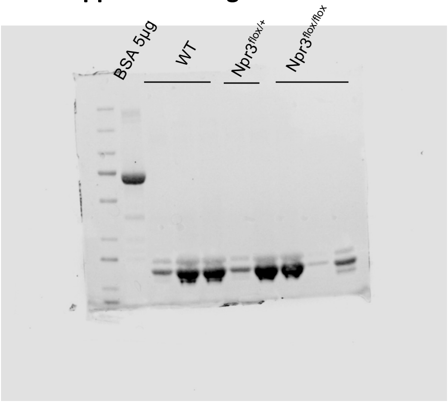

Supplemental Table 1

Primers used in the study

Gene	Forward	Reverse
**huNPR1**	ACGCATTGAGCTGACACGAAA	GATCCCAAAGCTGTATACGTCACC
**huNPR2**	CAGATTTGGTGGACGGGACG	TAGCCAGCTCCTTCTCCAGC
**huNPR3**	CAACCTTAGCAACGCCCAAA	GGAAGCGTCCTTCTCTGCAA
**mmNpr1**	CACTCAGCGGATGTGGAACC	CCATAATTGGAGCCTCGCCC
**mmNpr2**	CGCATTCGCTGGGAAGAACT	TCCCTTGAAGTGACCGGTGT
**mmNpr3**	GCTGTTCACTTTCTCGGCGT	AATCGTCCCGGGGCAATAGA
**hu29s**	TTGAAAATCCGGGGGAGAG	ACATTGTTCCAACATGCCAG
**mmGAPDH**	TGTTCCTACCCCCAATGTGT	TGTGAGGGAGATGCTCAGTG
**mmNPR3 exon 2**	CAGTGGAGACTACGCTTTCTTC	-
**mmNPR3 exon 5**	-	GTTGGCATCTATGGACACCTG
